# Assessment of risk factors associated with potential drug-drug interactions among patients suffering from chronic disorders

**DOI:** 10.1371/journal.pone.0276277

**Published:** 2023-01-24

**Authors:** Muhammad Fawad Rasool, Anees ur Rehman, Irfanullah Khan, Muhammad Latif, Imran Ahmad, Sadia Shakeel, Muhammad Sadiq, Khezar Hayat, Shahid Shah, Waseem Ashraf, Abdul Majeed, Iltaf Hussain, Rabia Hussain

**Affiliations:** 1 Department of Pharmacy Practice, Faculty of Pharmacy, Bahauddin Zakariya University, Multan, Pakistan; 2 Department of Clinical Pharmacy, School of Pharmaceutical Sciences, University Sains Malaysia, Penang, Malaysia; 3 Department of Zoology, Division of Science and Technology, University of Education, Lahore, Pakistan; 4 Department of Pharmaceutical Chemistry, Faculty of Pharmacy, Bahauddin Zakariya University, Multan, Pakistan; 5 Department of Pharmacy Practice, Faculty of Pharmaceutical Sciences, Dow University of Health Sciences, Karachi, Pakistan; 6 Institute of Pharmaceutical Sciences, University of Veterinary and Animal Sciences, Lahore, Pakistan; 7 Department of Clinical Pharmacy, Faculty of Pharmaceutical Sciences, Government College University, Faisalabad, Pakistan; 8 Department of Pharmacology, Faculty of Pharmacy, Bahauddin Zakariya University, Multan, Pakistan; 9 Department of Social and Administrative Pharmacy, School of Pharmaceutical Sciences, University Sains Malaysia, Penang, Malaysia; University Campus Bio-Medico di Roma, ITALY

## Abstract

Patients suffering from chronic diseases are more likely to experience pDDIs due to older age, prolonged treatment, severe illness and greater number of prescribed drugs. The objective of the current study was to assess the prevalence of pDDIs and risk factors associated with occurrence of pDDIs in chronic disease patients attending outpatient clinics for regular check-ups. Patients suffering from diabetes, chronic obstructive pulmonary disease (COPD), stroke and osteoporosis were included in the study. This study was a cross sectional, observational, prospective study that included 337 patients from outpatient clinics of respiratory ward, cardiac ward and orthopedic ward of Nishter Hospital Multan, Pakistan. The mean number of interactions per patient was 1.68. A greater risk for occurrence of pDDI was associated with older age ≥ 60 years (OR = 1.95, 95% CI = 1.44–2.37, p<0.001); polypharmacy (≥ 5 drugs) (OR = 3.74, 95% CI 2.32–4.54, p<0.001); overburden (OR = 2.23, 95% CI = 1.64–3.16, p<0.01); CCI score (OR = 1.28, 95% CI = 1.04–1.84, p<0.001); multiple prescribers to one patient (OR = 1.18, 95% CI = 1.06–1.41, p<0.01); and trainee practitioner (OR = 1.09, 95% CI = 1.01–1.28, p<0.01). Old age, polypharmacy, overburden healthcare system, higher comorbidity index, multiple prescribers to one patient and trainee practitioner were associated with increased risk of occurrence of pDDIs in chronic disease patients.

## 1. Introduction

Drug-drug interaction (DDI) is defined as an unwanted clinical effect of a drug under the influence of other drug resulting in compromised therapeutic efficacy or enhanced toxicity [1]. DDIs are associated with considerable economic burden to patients and healthcare system [2]. In the United States of America more people die due to DDIs than traffic accidents, auto immune deficiency Syndrome or breast cancer [3, 4]. DDIs are reported as 3rd leading cause of outpatient visits after cardiovascular diseases and cancer [5]. Globally 6.5% to 11.4% of all hospital admissions, and 22% of readmissions after discharge are due to drug related events [5]. In older patients’ hospital admissions due to DDIs are even higher, ranging from 10% to 19.6% [6]. Although the number of potential interacting drug combinations is very large only a small number are relevant in clinical practice. Potential drug-drug interactions (pDDIs) constitute one of the often-preventable cause of DDIs. Clinically significant pDDIs can result in increased outpatient visits, prolonged therapy, hospitalization, increased cost of treatment and higher mortality risk [7]. Approximately 46% of the hospital admissions due to pDDIs are preventable [6]. Avoiding pDDIs can result in substantial relief to healthcare system [8].

Previous studies reported the occurrence of pDDIs in prescriptions in range of 5% to 96%, irrespective of whether they lead to adverse drug reaction or not [9–11]. Difference in the burden of pDDIs among different studies is due to difference in study design, population included, the method utilized for the classification of the interactions, definition of pDDIs used and prescribing practice. Clinical risk factors for pDDIs include polypharmacy (i.e., ≥ five drugs), older age, hospital admissions, comorbidities and multiple prescribers [12]. In older patients (i.e., 65 years or above) the risk for pDDIs is reported as 73% higher as compared to young ones [6]. The risk of DDIs also increases with the increase in number of drugs [13]. The risk of pDDIs increases up to 30% in patients who received five or more drugs and 38% in patients with age greater than 75 years [13].

Chronic diseases (i.e., diabetes, COPD, stroke, and osteoporosis) cause substantial economic burden and are major cause of morbidity and mortality [14, 15]. Patents suffering from chronic diseases experience three times more economic burden than patients suffering from non-chronic diseases [16–18]. Patients suffering from chronic diseases are at increased risk to experience pDDIs due to multiple comorbidities, old age, polypharmacy, chronic therapeutic regimens, multiple outpatient visits, frequent hospital admissions, frequent modification in therapy, prolonged treatment and multiple prescribers [19]. Moreover, in a lower-middle income country like Pakistan lack of online drug interaction software in hospital settings to identify pDDIs, inappropriate prescribing, overburden healthcare system, lack of disease-surveillance system, and absence of clinical pharmacists in most of the settings can add additional risk for occurrence of pDDIs in chronic disease patients [20–22].

Knowledge about the risk factors of pDDIs can help to design and implement strategies to reduce the occurrence of pDDIs. In United States of America the economic burden due to drug related events reduced from US$ 177.4 billion in 2001 to US$ 21 billion in 2014 due to implementation of effective strategies to avoid pDDIs [4]. Similarly in Europe 40% reduction in pDDIs is reported due to implementation of effective strategies to report and resolve pDDIs [23]. Previous studies assessed different risk factors responsible for occurrence of pDDIs, but they were performed in specific populations, such as hospitalized patients, ICU admitted patients and patients suffering from specific diseases [2, 24, 25]. Few studies assessed the relationship between pDDIs and specific factors such as length of stay, mortality rate, patient age, number of drugs prescribed, multiple physician, review of prescription by a pharmacist or specialist and cost of hospitalization [12, 26–28]. Moreover, data on pDDIs is mostly available from hospitalized patients [24, 25]. To the best of our knowledge, no study assessed the incidence and causes of pDDIs in chronic disease (diabetes, COPD, stroke, and osteoporosis) patients visiting outpatient clinics for regular follow-up. Finding the factors associated with pDDIs in chronic disease patients can help healthcare professionals and policymakers to design and implement strategies to prevent pDDIs and improve management. Thus, the objective of the present study was to assess the prevalence of pDDIs and risk factors associated with occurrence of pDDIs in chronic disease patients attending outpatient clinics for regular check-ups. Patients suffering from diabetes, COPD, stroke and osteoporosis were included in the study [29].

## 2. Methodology

### 2.1. Study design

This study was a cross sectional, observational, prospective study that included 337 patients from outpatient clinics of orthopedic ward, respiratory ward and cardiac ward of Nishter Hospital Multan, Pakistan. The detailed methodology and inclusion criteria is already published somewhere else [30]. In brief patients were included in the study if (i) they had confirmed diagnosis of osteoporosis, diabetes, stroke and COPD (ii) prescription containing at least two drugs (iii) age ≥ 20 years. To avoid biasness data was collected from every 3rd patient visiting the outpatient clinic. Data collection was done during December 2017 to August 2018. The research protocol was approved by the Research and Ethics committee of Faculty of Pharmacy Bahauddin Zakariya University, Multan Pakistan and the concerned hospital (registration no. Bzbp-18-4207). Written informed consent was obtained from all participants.

### 2.2. Data collection

Data collectors (graduating class pharmacy students) were recruited for data collection and trained by an academic investigator. Data was collected on a self-developed questionnaire. Collected data include patient’s demographic data (i.e., age, sex), socioeconomic status, disease, diagnosis, medication (i.e., drug names, dosage and frequency of prescribed drugs), self-medication if any, physician specialization, comorbidities, and number of prescribing physicians for different diagnosis. The data collection form was completed after physician consultation.

Polypharmacy patient was defined as a patient utilizing ≥ 5 drugs [28]. Overburden was considered to check 20 or more patients in an hour. Charlson comorbidity index (CCI) was used to calculate comorbidity burden [31]. The CCI evaluates the comorbidity burden by weighing and summing the patient reported conditions (e.g. peptic ulcer, dementia, myocardial infarction, peripheral vascular diseas, CHF, diabetes mellitus, chronic kidney disease, connective tissue disease, hemiplegia, cancer, COPD, lymphoma, leukaemia, AIDS and cerebrovascular accident). Higher score indicates greater comorbidity burden.

### 2.3. Potential drug-drug interactions

Drug interaction software; Micromedex^®^ (Thomson Reuters Healthcare Inc., Greenwood Village, Colorado, United States) was used to assess the pDDIs in the prescriptions [32]. For drugs containing two or more active ingredients, each active ingredient was treated as separate drug. Each active ingredient was added separately in the software to check the interactions. All pDDIs were displayed in the output display of the Micromedex^®^. Micromedex classifies the interactions as minor interactions (may cause increase in side effects), moderate interactions (require change in therapy or monitoring), major interactions (may have a risk of any organ damage) and contraindicated interactions (strictly prohibited and may be life threatening) [33].

## 3. Data analysis

Frequencies and percentages were reported for categorical variables and the mean and standard deviation for continuous variables. Normality of the data was checked using Shapiro–Wilk test of normality. Characteristics of patients in different groups stratified according to presence or absence of pDDIs were compared using independent-samples t-test for continuous variables and Chi-square test for categorical variables. Univariate and multivariate logistic regression analysis were used to assess the impact of old age, gender, polypharmacy, CCI score, trainee practitioner, prescription by a specialist, multiple prescribers, involvement of clinical pharmacist, presence of medical record, use of online softwares and overburden on occurrence of pDDIs in patients suffering from diabetes, COPD, stroke or osteoporosis. Initially a univariate analysis was performed and variables with univariate p values ≤0.02 were included in multivariate regression model. Odds ratio was used to assess the influence of different risk factors. The Hosmer–Lemeshow test was used to check goodness-of-fit of the model. A p value of 0.01 or less was considered statistically significant. All analysis were performed using SPSS version 24.0 (IBM SPSS Statistics for Windows, 142 Armonk, NY: IBM Corporation).

## 4. Results

Out of 337 patients 40.95% were male with mean age 48.34 (9.4) years. Among the included patients 34.72% were suffering from Diabetes mellitus, 15.13% were suffering from COPD, 20.18% were suffering from Stroke, 42.73% were suffering from Hypertension and 18.10% were suffering from Osteoporosis. The average number of medicines per encounter was 5.23 (2.8). More than five drugs were prescribed in almost 73.29% patients. 31.27% of patients had multiple prescriptions dispensed by more than one department on the same day. When stratified according to presence or absence of potential pDDIs, significant differences were observed in age, no. of prescribed drugs, polypharmacy (≥5 drugs), no. of prescribing physicians, no. of comorbidities and CCI scores among the patients. The demographic and clinical data of patients according to presence or absence of pDDIs are shown in Table 1.

**Table 1:** Demographic and Clinical characteristics of the patients included in the study (N = 337).

Variables	All Patients (n = 337)	Showing pDDIs (n = 220)	No pDDIs (n = 117)	p value^a^
Gender, male	138 (40.95%)	107 (48.64%)	31 (26.50%)	0.19
Mean age	48.34 (9.4)	58.82 (10.3)	35.21 (9.1)	<0.01
Patients age ≥60 years	131 (38.87%)	95 (43.18%)	36 (30.76%)	<0.001
Mean no. of prescribed drugs	5.23 (2.8)	7.56 (3.3)	3.14 (2.8)	<0.01
Patients prescribed with ≥ 5 drugs	247 (73.29%)	194 (88.18%)	53 (45.29%)	<0.01
Mean no. of physicians who prescribed to single patient	1.47 (0.73)	1.98 (1.02)	1.05 (0.68)	<0.01
Mean no. of diseases per patient	1.8 (0.81)	2.74 (0.91)	1.2 (0.56)	<0.01
Mean CCI	1.73 (1.44)	2.65 (1.46)	0.73 (0.34)	<0.001
CCI≥2	135 (40.06%)	121 (55%)	14 (11.96%)	<0.01
**Diagnosis** ^b^				
Diabetes mellitus	117 (34.72%)	89 (40.45%)	28 (23.93%)	<0.01
COPD	51 (15.13%)	42 (19.09%)	9 (7.69%)	<0.01
Stroke	68 (20.18%)	55 (25%)	13 (11.11%)	<0.01
Hypertension	144 (42.73%)	112 (50.91%)	32 (27.35%)	0.18
Osteoporosis	61 (18.10%)	49 (22.27%)	12 (10.26%)	0.02

Results are presented as mean (standard deviation) and number (percentage) unless otherwise stated;

^a^The difference was assessed among groups stratified according to presence or absence of pDDI; pDDI, Potential drug-drug interaction;

^b^ the sum may be greater than 337 as some patients were suffering from more than one disease; CCI, Charlson comorbidity index; COPD chronic obstructive pulmonary disease.

A total 220 (65.28%) prescriptions showed pDDIs. The mean number of interactions per patient was 1.68 (0.8). Among the prescription with pDDIs 164 (74.55%) prescriptions showed one or two and 56 (25.45%) prescriptions showed three or more than 3 drug-drug interaction regardless of severity. Among drug-drug interactions 16 (4.34%) were contraindicated, 67 (18.16%) were severe, 172 (46.61%) were moderate, and 114 (30.89%) were minor. Prevalence of pDDIs in the prescriptions and patients are presented in Table 2.

**Table 2:** Prevalence of potential drug-drug interactions in chronic disease patients.

**Potential Drug-Drug Interactions (pDDIs)**	
Prescriptions with pDDIs	220 (65.28%)
Prescriptions with 2 pDDIs	164 (74.55%)
Prescriptions with 3 or more pDDIs	56 (25.45%)
Total pDDIs	369
Minor pDDIs	114 (30.89%)
Moderate pDDIs	172 (46.61%)
Severe pDDIs	67 (18.16%)
Contraindicated pDDIs	16 (4.34%)
**Mean pDDIs per patient**	
Overall	1.68 (0.8)
Minor pDDI	0.46 (1.96)
Moderate pDDI	0.67 (2.03)
Severe pDDI	0.19 (0.3)
Contraindicated pDDI	0.07 (.39)

Results are presented as mean (standard deviation) and number (percentage) unless otherwise stated; Overall prevalence, presence of atleast one pDDI regardless of type and severity.

Independent variables showing significant relationship with pDDIs in univariate analysis (p<0.01) were added in the multivariable logistics regression. The odd ratios (OR), 95% confidence intervals and p values for the independent variables included in the univariate and multivariable logistic regression are represented in Table 3. In multivariable logistic regression, greater risk for occurrence of pDDI was associated with older age ≥ 60 years (OR = 1.95, 95% CI = 1.44–2.37, p<0.001); polypharmacy (≥ 5 drugs) (OR = 3.74, 95% CI 2.32–4.54, p<0.001); overburden (OR = 2.23, 95% CI = 1.64–3.16, p<0.01); CCI score (OR = 1.28, 95% CI = 1.04–1.84, p<0.001); multiple prescribers to one patient (OR = 1.18, 95% CI = 1.06–1.41, p<0.01); and trainee practitioner (OR = 1.09, 95% CI = 1.01–1.28, p<0.01).

**Table 3:** Multiple logistic regression analysis for risk factors associated with pDDIs in chronic disease patients.

	Univariate logistic regression (N = 220)			Multivariable logistic regression (N = 220)		
Variables	OR	95% CI	P value	OR	95% CI	p value
Gender, male	0.88	0.31–1.23	0.11	0.72	0.52–1.08	0.28
Older age ≥ 60 years	2.17	1.32–3.11	<0.001	1.95	1.44–2.37	<0.001
Polypharmacy (≥ 5 drugs)	4.94	2.26–6.67	<0.001	3.74	2.32–4.54	<0.001
Overburden ^a^	3.02	1.63–5.76	<0.01	2.23	1.64–3.16	<0.01
CCI score	1.87	1.13–3.07	<0.001	1.28	1.04–1.84	<0.001
multiple prescribers to one patient	1.51	1.27–2.13	<0.001	1.18	1.06–1.41	<0.01
trainee practitioner	1.32	1.09–1.77	<0.01	1.09	1.01–1.28	<0.01
prescription by a specialist	0.88	0.53–0.98	<0.01	0.83	0.55–1.17	0.09
presence of previous medical record	0.73	0.49–0.95	<0.01	0.63	0.37–1.24	0.16
use of online software in prescription generation	0.69	0.51–0.86	<0.01	0.66	0.45–1.22	0.15
review of prescription by clinical pharmacist	0.63	0.34–0.88	<0.01	0.58	0.27–0.89	0.37

OR, odd ratio; p <0.01 was considered statistically significant;

^a^ ≥20 patients in one hour; CCI, charlson comorbidity index.

The association was not significant with patient’s gender (OR = 0.72, 95% CI = 0.52–1.08, p = 0.28); presence of previous medical record (OR = 0.63, 95% CI = 0.37–1.24, p = 0.16); use of online software in prescription generation (OR = 0.66, 95% CI = 0.45–1.22, p = 0.15); prescription by a specialist (OR = 0.83, 95% CI = 0.55–1.17, p = 0.09); and review of prescription by a clinical pharmacist (OR = 0.58, 95% CI = 0.27–0.89, p = 0.37).

The model explained the variance (accuracy) of 64.73, with Hosmer-Lemeshow tests showing no significant differences (p = 0.914) and Goodness of Fit Model showing significant difference (p<0.01) between observed and predicted probabilities.

## 5. Discussion

Drug-drug interactions result in increasing burden on health care system. Chronic disease patients are at increased risk of occurrence of pDDIs. But its still confusing which factors are best predictors of pDDIs in chronic disease patients. This study assessed the association of different factors with increased risk of occurrence of pDDIs in chronic disease patients. Older age, polypharmacy (≥ 5 drugs), overburden healthcare system, higher comorbidity index, multiple prescribers to one patient and trainee practitioner were associated with increased risk of occurrence of pDDIs in chronic disease patients. Our results are in line with the previous research. In a study from Serbia, Kovačević et al. assessed the prevalence of pDDIs in hospital admitted cardiovascular patients. At least one potentially relevant pDDI was identified in 83.9% of patients. Patients with longer hospital stay, polypharmacy and comorbidities were at increased risk of pDDIs [24]. Roblek et al. assessed the pDDIs in hospitalized chronic heart failure (CHF) and COPD patients [34]. Patients with CHF showed more pDDIs than patients with COPD. Patients with concomitant CHF and COPD showed more pDDIs than patients with any disease alone. They reported that prescriptions given on discharge from hospital contain more pDDIs than prescriptions given during hospital admission. Moreover, the number of pDDIs increased in patients with concomitant CHF and COPD [34]. Bacic-Vrca et al. assessed the prevalence of pDDIs in arterial hypertension patients in a community setting [35]. On average each patient was utilizing five drugs and 83.3% patients reported at least one potential DDI in their prescription [35]. The difference in prevalence of pDDIs in different countries, may be due to different DDI data bases, different prescribing practices and study population.

Age and number of prescribed drugs showed significant association with the increased risk of pDDIs in chronic disease patients. Previous studies reported substantial burden of pDDIs in old age patients [19, 36, 37]. Oscar et al reported 82.13% prevalence of pDDIs in old age patients among which 43.56% were severe in nature [37]. Milosavljevic et al. assessed the prevalence of pDDIs in surgical patients. Number of prescribed drugs, comorbidities and number of physicians who prescribed to one patient were significantly associated with increase in the risk of pDDIs [25]. Santos-Díaz et al. assessed the prevalence and risk factors for occurrence of pDDIs in chronic kidney disease (CKD) patients. Age and number of drugs were significantly associated with increase in the risk of pDDIs in CKD patients [38]. Lynskey et al. and Kovačević et al. demonstrated that the incidence of pDDIs in all age patients increased with the addition of every single drug to the prescription. Polypharmacy was considered as utilization of 5 or more drugs. Substantial number of patients were prescribed with five or more drugs. Our results showed significant association of polypharmacy with pDDIs. This is inline with the previously published research. Literature review shows that increase in the number of drugs in a prescription, increases the risk of pDDIs [2, 39]. Weideman et al., reported 66% increase in the risk of pDDIs in prescriptions with more than five drugs as compared to less than five drugs [40]. Polypharmacy requires special attention of the healthcare professional. Drugs with frequent ADRs and narrow therapeutic window should be monitored closely when in use.

Comorbidities were associated with increased risk of pDDIs due to increase in number of medications. CCI can assess the comorbidity risk. Comorbidities were weighed on scale to generate the CCI score. CCI score was associated with the increase in risk of medication errors. The risk of medication errors increased 1.87 time in patients with CCI score greater than 2 as compared to patients with CCI score less than 2. Our results are in line the study of Arabyat et. al, who demonstrated the significant association of higher CCI score and medication error [41]. In a previous research presence of diabetes and hypertension and COPD were associated with increase in the risk of pDDIs [25]. Chronic diseases are treated with combination of drugs, which have a notable potential to interact with other drugs [42]. Most of the interactions were due to improper implementation of treatment guidelines than irresponsible clinical practice [34]. Better guideline implementation may result in implementation of most of the interactions. Comorbidities enhance the physician visits of the patients and usage of medicines. Sometimes, patients need to see multiple physicians at the same time for different diagnosis [19]. In a country like Pakistan medical records of the patients are not maintained. Absence of medical records may enhance the risk of pDDIs [19]. Overburden was also associated with the elevated risk of pDDIs. Overburden results in compromised efficiency of healthcare professional to identify pDDIs. Optimum workload may have positive effects in avoiding pDDIs. Previous research showed the positive impact of optimum workload in avoiding pDDIs [43].

Ability to recognize pDDIs is important to reduce the harms associated with pDDIs on patients and healthcare system [44]. In the presence of hundreds of drugs it is difficult to remember all the pDDIs [45, 46]. So aiding the prescription process with different means like online software to check pDDIs, review by a senior physician or clinical pharmacist may help to reduce the risk of pDDIs [47]. Information technology can be used to learn about pDDIs and to get access to patient medical records [48]. Research shows that digitalization of healthcare system can reduce the chances of errors. Physicians aided with personal digital assistance and online system to check pDDIs were less prone to prescribing errors [44, 49]. Another study showed that the use of online software programs during prescribing process reduced the chances of pDDIs up to 66% in general practice [50].

Our results showed that specialization in a specific disease reduced the risk of pDDIs. Disease specialists usually prescribe specific categories of drugs. They are more familiar with the interactions of these specific drugs. More, frequent prescribing of same drugs reduces the chances of pDDIs due to enhanced clinical experience. Our findings are in line with the previous literature. Ko Y et al. demonstrated that clinical experience reduces the chances of pDDIs [47]. Another study demonstrated that prescriptions from general practitioners were more prone to medication errors than prescriptions from disease specialists [51]. Clinical training of junior medical staff and feedback control of prescribing by therapeutic committee may also reduce the chances of pDDIs [52]. Moreover, implementation of approved disease guidelines may also reduce the risk of pDDIs [45, 52].

The review of prescription by a clinical pharmacist also showed no significant association with the occurrence of pDDIs. In addition to online software programs, pharmacists are considered as most widely used information source to identify pDDIs [47]. Zaal et al. in a longitudinal prospective study, reported that review of the prescription by a clinical pharmacist effectively reduced pDDI by identifying interactions which were not detected during routine clinical practice [53]. The importance of clinical pharmacist in healthcare system is well recognized but unfortunately in Pakistan the role of clinical pharmacist is not well-established. In Germany, implementation of pharmaceutical care services in cardiology departments resulted in reduced drug related events, less readmission and improved management [54]. Literature shows that the involvement of clinical pharmacist in healthcare system reduced the risk of pDDIs, increased adherence to therapy and support physician to manage adverse drug events due to pDDIs [53, 55].

This study was conducted in specific population (chronic disease patients), so its results should be generalized with caution. But it can be assumed that the results found in this study can be generalized to chronic disease patients. Another limitation of the study is only pDDIs were studied. Patients were not followed-up to assess the rate of adverse drug events due to pDDIs. Most of the minor and moderate pDDIs can be managed with increasing the duration of interval between administrations of drug or adjusting dose [24]. So it was not clear how many pDDI exactly lead to adverse drug events.

## 6. Conclusion

Chronic disease patients are at increased risk of occurrence of pDDIs. The mean number of interactions per patient was 1.68. Among drug-drug interactions 4.37% were contraindicated, 18.16% were severe, 46.61% were moderate, and 30.89% were minor. Old age, no. of drugs prescribed, overburden healthcare system, higher comorbidity index, multiple prescribers to one patient and trainee practitioner were associated with increased risk of occurrence of pDDIs in chronic disease patients. Whereas, gender, presence of previous medical record, use of online software in prescription generation, prescription by a specialist and review of prescription by a clinical pharmacist showed no significant association with occurrence of pDDIs in chronic disease patients. Most of the pDDIs are preventable. Ability to recognize pDDIs is important to reduce the harms associated with pDDIs on patients and healthcare system. So aiding the prescription process with different means like online software to check pDDIs, review by a senior physician or clinical pharmacist may help to reduce the risk of pDDIs.
